# Some Cranial Surgery

**Published:** 1893-10

**Authors:** Thomas J. Pugh

**Affiliations:** Hearne, Texas


### Correspondence.

### SOME CRANIAL SURGERY.

HEARNE, TEXAS, July 28, 1893.

*Editor Texas Medical Journal:*

DEAR SIR:—During a period of twenty-three years' practice of medicine, I have performed the operation of trephining and raising depressed cranial bones three times. First operation in November, 1874, at Mudville, Brazos county, Texas. The second operation, December, 1880, near same place. The third operation near Hearne, Texas, on or about June 10, 1893.

The first operation was a perfect success. The patient, a negro some forty years of age, was struck with the back of a heavy ax, on the left parietal bone, crushing and mashing the bone down on the brain. The patient remained in an unconscious condition till after operation, which was performed some twelve hours after the injury. Consciousness was immediately restored after the depressed bones were removed, and the man made speedy recovery, and has made a good plantation hand since.

The second case, a boy some ten years old, was kicked by a mule over the left eye, mashing in about one-half of frontal bone. Trephining and elevating depressed bones were done some ten hours after injury. The patient rallied well, and gave promise of recovery, but after three weeks, died.

The third case operated upon was performed near Hearne, on Mr. J. E. Kirby, an old gentleman sixty-nine years of age. Some time during the early part of June, 1893, about the 10th, I think, Mr. Kirby had trouble with one of his tenants, upon whom he advanced with a drawn knife. The assailed party struck Mr. Kirby three severe blows on the head with the cutting surface of a hatchet. One cut was on the frontal bone,—a long, ugly wound, which did not penetrate the bone,—the other two licks were made in the right and left parietal bones, near their junction with the occipital bone. The wounds penetrated into the brain substance, as evidenced by flowing brain tissue through the wounds. The patient was not entirely unconscious, yet his wounds were grave, and I informed his wife and friends that without an operation he must soon die, and with an operation his chance for recovery was indeed slim. The wife permitted me to operate, and assisted by Dr. C. T. Doremus, of Hearne, I proceeded to raise the depressed bone, all of which was accomplished in about half an hour. The operation was done *without* the antiseptic regime, save the free use of pure cold water.

I have no confidence in the use of this now much vaunted and ridiculous preparation with instruments boiled in bichloride solution and well washed and scrubbed integument. I have done some nice amputations in my past twenty-three years' practice, and have done the three above mentioned cranial operations, and I have had no reason to regret having used my instruments just as I took them from my case.

But to return to my last case of cranial surgery; three weeks after the operation the patient, Mr. Kirby, geared up his wagon and drove into town, some three miles distant from his home. The old man, now some five weeks after the operation, says he is well and all right.

THOMAS J. PUGH, M. D.

**College of Physicians of Philadelphia**, Northeast corner of Thirteenth and Locust streets. The William F. Jenks memorial prize, the third triennial prize of five hundred dollars, under the deed of trust of Mrs. William F. Jenks, will be awarded to the author of the best essay on "INFANT MORTALITY DURING LABOR, AND ITS PREVENTION."

The conditions annexed by the founder of this prize are, that the "prize or award must always be for some subject connected with Obstetrics, or the Diseases of Women, or the Diseases of Children;" and that "the trustees, under this deed for the time being, can, in their discretion, publish the successful essay, or any paper written upon any subject for which they may offer a reward, provided the income in their hands may, in their judgment, be sufficient for that purpose, and the essay or paper be considered by them worthy of publication. If published, the distribution of said essay shall be entirely under the control of said trustees. In case they do not publish the said essay or paper, it shall be the property of the College of Physicians of Philadelphia."

The prize is open for competition to the whole world, but the essay must be the production of a single person.

The essay which must be written in the English language, or if in a foreign language, accompanied by an English translation, should be sent to the College of Physicians of Philadeplhia, Pennsylvania, U. S. A., before January 1, 1895, addressed to Horace Y. Evans, M. D., Chairman of the William F. Jenks Prize Committee.

Each essay must be typewritten, distinguished by a motto, and accompanied by a sealed envelope bearing the same motto and containing the name and address of the writer. No envelope will be opened except that which accompanies the successful essay.

The committee will return the unsuccessful essays if reclaimed by their respective writers, or their agents, within one year.

The committee reserves the right not to make an award if no essay submitted is considered worthy of the prize.

JAMES V. INGHAM,

Secretary of the Trustees.

August 1, 1893.

